# Induced Mesenchymal Stem Cells: An Emerging Source for Regenerative Medicine Applications

**DOI:** 10.3390/jcm14062053

**Published:** 2025-03-18

**Authors:** Mahmood S. Choudhery, Taqdees Arif, Ruhma Mahmood, Asad Mushtaq, Ahmad Niaz, Zaeema Hassan, Hamda Zahid, Pakeeza Nayab, Iqra Arshad, Mehak Arif, Mashaim Majid, David T. Harris

**Affiliations:** 1 Department of Human Genetics & Molecular Biology, University of Health Sciences, Lahore 50161, Pakistan; ms20031@yahoo.com (M.S.C.); taqdeesarif01@gmail.com (T.A.); asadmushtaq208@gmail.com (A.M.); ahmadniaz0189@gmail.com (A.N.); zaeemahassan123@gmail.com (Z.H.); zahidhamda315@gmail.com (H.Z.); nayabpakeeza2@gmail.com (P.N.); iqraarshad2204@gmail.com (I.A.); mehakarif313@gmail.com (M.A.); mashaimmajid17@gmail.com (M.M.); 2 Department of Pediatric Surgery, Allama Iqbal Medical College, Jinnah Hospital, Lahore 54700, Pakistan; ruhma_mahmood@yahoo.com; 3 Department of Immunobiology, University of Arizona Health Sciences Biorepository, College of Medicine, University of Arizona, Tucson, AZ 85721, USA

**Keywords:** mesenchymal stem cells, induced pluripotent stem cells, induced mesenchymal stem cells, regenerative medicine

## Abstract

Regenerative medicine is gaining interest in the medical field due to the limitations of conventional treatments, which often fail to address the underlying cause of disease. In recent years, stem cell-based therapies have evolved as a promising alternative approach to treat those diseases that cannot be cured using conventional medicine. Adult stem cells, particularly the mesenchymal stem cells (MSCs), have attracted a lot of attention due to their ability to regenerate and repair human tissues and organs. MSCs isolated from adult tissues are well characterized and are currently the most common type of cells for use in regenerative medicine. However, their low number in adult donor tissues, donor-age and cell-source related heterogeneity, limited proliferative and differentiation potential, and early senescence in in vitro cultures, negatively affect MSC regenerative potential. These factors restrict MSC use for research as well as for clinical applications. To overcome these problems, MSCs with superior regenerative potential are required. Induced MSCs (iMSCs) are obtained from induced pluripotent stem cells (iPSCs). These cells are patient-specific, readily available, and have relatively superior regenerative potential and, therefore, can overcome the problems associated with the use of primary MSCs. In this review, the authors aim to discuss the characteristics, regenerative potential, and limitations of MSCs for regenerative medicine applications. The main methods to generate iMSCs from iPSCs have been discussed in detail. In addition, the proposed criteria for their molecular characterization, applications of iMSCs for disease modeling and drug discovery, as well as potential use in regenerative medicine have been explored in detail.

## 1. Introduction

Cell-based therapy was initially employed in the 1950s, when hematopoietic stem cells (HSCs) from a twin were utilized to regenerate blood cells of a leukemia patient. Since then, stem cell-based therapy has evolved as a promising alternative approach to treat diseases, particularly those that traditional medicines cannot treat. Stem cell-based therapy utilizes unique properties of stem cells (i.e., self-renewal, and differentiation into multiple tissues) to repair, regenerate, or replace damaged cells and tissues in the body [1]. Stem cells are broadly categorized into embryonic stem cells (ESCs) and adult stem cells. ESCs are pluripotent stem cells as they can differentiate into all cell types in the body. However, the therapeutic use of ESCs is controversial due to ethical, legal, and religious concerns. Adult stem cells are found in almost all organs and tissues of the body, where they are involved in the repair, replacement, and regeneration of damaged tissue and organs. Mesenchymal stem cells (MSCs), a type of adult stem cells, are of particular interest for regenerative medicine applications because of their repair, angiogenic, and immunomodulatory properties [2]. These cells exhibit remarkable ability to differentiate into multiple cell types. MSCs can also modulate the immune response by suppressing the proliferation of immune cells (such as T cells, B cells, natural killer cells) and by inducing regulatory T cells, thereby mitigating inflammation and promoting tissue repair. Results of preclinical and clinical studies exhibit potential of MSCs to treat various diseases including cardiovascular, neurological, autoimmune, and musculoskeletal. Despite the promising therapeutic potential of MSCs, certain challenges restrict their clinical use [3,4,5]. Limitations associated with clinical use of MSCs include a low number in adult tissues, donor-to-donor variations in terms of number and potential of MSCs, reduced proliferative and differentiation potential with increased donor age, apoptotic and senescence features in in vitro cultures, a lack of standardized isolation and expansion protocols, etc. These constraints limit the therapeutic utility of MSCs. Therefore, it is imperative to address these challenges for better clinical outcomes [3,4,5].

Induced pluripotent stem cells (iPSCs) are generated by the reprogramming of adult somatic cells, thereby rendering them to an undifferentiated embryonic-like state. These cells can differentiate into any specific cell type found in the human body. In addition, iPSCs have the ability to continuously renew themselves without the signs of senescence. Since iPSCs can be cultured continuously for extended periods, they represent a non-exhausted source of cells for producing other types of cells in the body. Interestingly, iPSCs can be exploited to generate iMSCs. iMSCs are a type of MSCs that can be generated from iPSCs through a process of directed differentiation. They possess characteristics similar to those of native MSCs, including the ability to differentiate into various cell types, such as osteocytes, chondrocytes, and adipocytes. Many such methods have recently been devised to produce iMSCs from iPSCs. Each method to produce iMSCs from iPSCs, however, has its own advantages and limitations. Despite the intrinsic ability of iPSCs to differentiate independently into cells of all three lineages, it is important to carefully analyze the consistent production of iMSCs at large-scale and to ensure reliability of methods employed. The consistent production of iMSCs from iPSCs has the potential to overcome the limitations of primary MSCs and will be effectively scaled up for commercial applications. This review discusses the properties, applications, and limits of primary MSCs for regenerative medicine applications, with subsequent sections focusing on iMSCs as a promising alternative to native MSCs. The most common methods/protocols for generating iMSCs from iPSCs have been described. Furthermore, proposed molecular characterization criteria, iMSC applications for disease modeling and drug development, and prospective application in regenerative medicine have been thoroughly investigated.

## 2. Overview of Application of MSCs

Mesenchymal stem cells (MSCs) are an important source of cells for regenerative medicine applications. MSCs have high proliferative potential and ability to differentiate into other cell types that originate from the mesoderm, including adipocytes, osteocytes, and chondrocytes. MSCs are promising therapeutic agents for the treatment of a wide range of disorders due to their unique regenerative, angiogenic, and inflammatory properties. MSCs also have the potential to produce an immunomodulatory environment through the production of soluble factors. MSCs can be isolated from various tissues and organs, several of which are readily available for use. Results of clinical and pre-clinical studies suggest that MSCs are a valuable therapeutic source for a range of disorders. MSCs are preferred due to their unique therapeutic qualities, as well as their ease of accessibility and expansion. Currently, MSCs have been successfully applied for orthopedic injuries, graft versus host disease, cardiovascular diseases, autoimmune diseases, liver diseases, skin diseases, etc. Additionally, MSCs that have been genetically modified to overexpress antitumor genes show promise as a cancer treatment [6].

Multiple clinical studies using MSCs have shown positive therapeutic outcomes. Currently 1699 clinical trials have been registered using MSCs for various conditions. A summary of the registered clinical trials was obtained from the www.clinicaltrials.gov website, accessed on 25 January 2025. The following searches were performed: “MSC-based clinical trials” OR “MSC therapy”. These trials have been classified into different statuses including not yet recruiting (*n* = 104), recruiting (*n* = 222), active but not recruiting (*n* = 77), completed (*n* = 581), terminated (*n* = 66), enrolling by invitation (*n* = 7), suspended (*n* = 24), withdrawn (*n* = 71), and unknown (*n* = 520). A significant number of these trials focus on cardiovascular disorders, with over 223 studies evaluating the therapeutic potential of MSCs in conditions such as ischemic cardiomyopathy. Similarly, neurodegenerative diseases, such as Parkinson’s disease and Alzheimer’s, have been the subject of approximately 84 trials. There have been approximately 54 clinical trials registered for kidney diseases, including acute renal failure and chronic kidney disease, 148 trials for osteoarthritis, and 109 trials for skin disorders. These trials suggest that MSCs are of particular interest for the treatment of a variety of diseases and disorders.

## 3. Limitations of Primary MSCs

**Figure 1:**
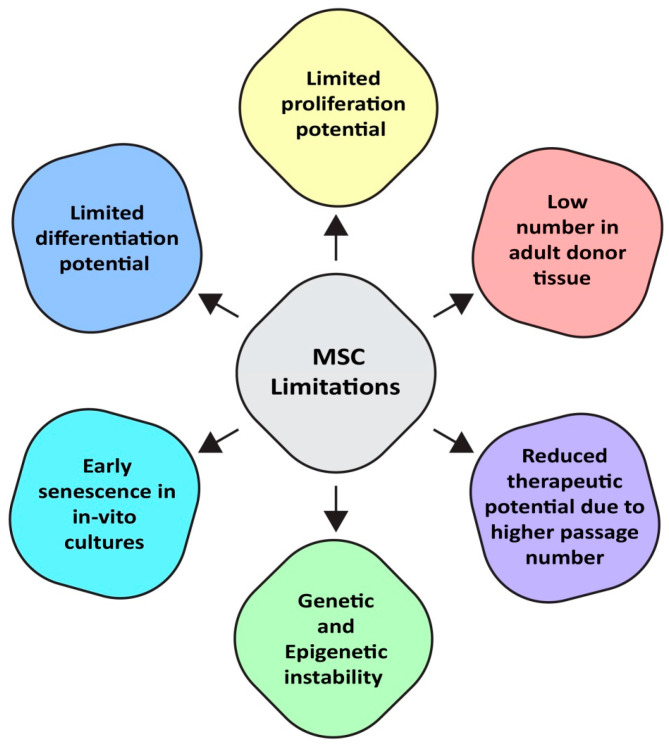
Limitations of primary mesenchymal stem cells. MSCs have a lower number in adult tissues, reduced therapeutic potential, genetic and epigenetic instability, limited proliferation and differentiation potential, early senescence in in vitro cultures, and risk of tumorigenesis after stem cell-based therapy.

Despite the potential of MSCs, there are a number of challenges that limit the use of MSCs for clinical purposes. Low number of MSCs in adult donor tissues, donor-age and cell-source related heterogeneity, limited proliferative and differentiation potential, and early senescence in in vitro cultures negatively impact their regenerative potential (Figure 1). In addition, the harvesting procedures of MSCs from different sources can be invasive, particularly from bone marrow. Bone marrow aspiration involves insertion of a large-bore needle in the iliac crest (hipbone). The procedure may cause pain, infection risk, and other complications. Another challenge related to the success of clinical applications of MSCs is the procurement of sufficient cell numbers. MSCs are low in numbers in tissues and are therefore expanded in in vitro cultures to obtain the required MSC number. However, the effects of passage number negatively influence the therapeutic potential and biological properties of MSCs. In prolonged culture, the potential of MSCs declines. Their regenerative potential is negatively impacted due to the reduced proliferation and differentiation. Similarly, the yield of MSCs varies among different sources of adult tissues. For example, MSCs derived from bone marrow contain 500-fold less MSCs than adipose tissue [7]. Similarly, umbilical cords yield a substantial number of cells. Aged people are the main population of patients for cell therapy. However, donor age is inversely proportional to the number of MSCs [8]. Moreover, the MSC number is significantly reduced in individuals with compromised health [9]. Over time, primary MSCs experience senescence due to various interconnected biological processes, which reduces their ability to divide and maintain their multipotent capacity. This is specifically challenging when large numbers of cells are needed for therapeutic usage [10]. Primary MSCs are liable to be deprived of their stem cell properties during in vitro growth [11]. It is important to address all the challenges before the full potential of MSCs can be utilized in clinical settings. These challenges highlight the need for novel MSC sources that are accessible in therapeutic dosages, without the problems associated with primary MSCs, and are suitable for use in clinical settings. One of such sources to generate MSCs, i.e., iMSCs, is induced pluripotent stem cells (iPSCs).

## 4. Overview of Pluripotent Stem Cells

Pluripotent stem cells (PSCs) are defined by their ability to differentiate into any type of cell in the body. This includes cells from all three germ layers (ectoderm, mesoderm, and endoderm) and germ line cells that help produce sperm and eggs [12]. These cells are pivotal in advancing regenerative medicine, disease modeling, and drug discovery. Pluripotent stem cells consist of two types, i.e., embryonic stem cells (ESCs) and induced pluripotent stem cells (iPSCs). ESCs originate from the inner cell mass (ICM) of a developing embryo. These cells are, however, controversial due to several ethical, religious, and legal concerns. The process of reprogramming or dedifferentiating adult somatic cells in vitro yields iPSCs. iPSCs were first generated in 2006 by Shinya Yamanaka and Kazutoshi Takahashi at Kyoto University [13]. They reprogrammed mouse fibroblasts into iPSCs using four transcription factors, namely, octamer-binding transcription factor 4 (Oct4), sex-determining region Y-box 2 (Sox2), cellular myelocytomatosis oncogene (c-Myc), and Kruppel-like factor 4 (Klf4), also known as Yamanaka factors [14]. These factors induce a complex series of genetic and epigenetic modifications and thus regulate pluripotency. The reprogramming strategies can be integrative or non-integrative (episomal vectors). Integrative reprogramming strategies involve the introduction of reprogramming factors into somatic cells via genome-integrating vectors (such as lentiviral vectors), which results in stable and permanent genomic modifications. Whereas non-integrative reprogramming strategies avoid permanent genomic modifications and are preferable for iPSC generation and more suitable for clinical applications. iPSCs have pivotal roles in both clinical and therapeutic applications. iPSCs can be developed from somatic cells of patients and hence can be used for disease modeling. They may also offer a personalized treatment with possibly a low chance of immunological rejection as the reprogrammed cells can be patient specific [15]. The ability to transform differentiated somatic cells into any type of adult cell is made possible with iPSCs. These cells can be induced to differentiate into MSCs called iMSCs (induced MSCs). iMSCs can overcome the limitations of primary MSCs. iMSC generation from iPSCs combines the advantages of MSC regenerative capabilities with the pluripotent nature of iPSCs, which results in an unlimited source of patient-specific cells for cell therapy. Various methods to generate iMSCs from iPSCs have been discussed in detail in Section 6.

## 5. iMSCs: A New Horizon for Regenerative Medicine Applications

The ability of the iPSCs to generate iMSCs represents a promising avenue for regenerative medicine applications. Availability in large numbers as well as their potential makes iMSCs ideal candidates for cell-based therapies. iMSCs share several characteristics with primary MSCs, such as multipotency, high proliferation, immunomodulation, repair, and regeneration potential. Moreover, iMSCs derived from iPSCs offer abundance, good efficacy, higher homogeneity, and address concerns of immune rejection and ethical issues [16]. The comparisons of donor-matched iMSCs and primary MSCs show that iMSCs might have enhanced proliferation potential and improved paracrine functions with more potent immune regulatory effects [17]. iMSCs may represent a practical cell type for regenerative medicine and tissue engineering applications [18]. iMSCs can differentiate into various cell types, including osteocytes, chondrocytes, and adipocytes, making them ideal for treating various diseases. As iMSCs can be produced in large numbers from iPSCs, they represent a limitless alternative supply of MSCs. Due to their encouraging therapeutic potential in both preclinical and clinical settings, iMSCs are a new alternative source of cells [19].

iMSCs have the potential to revolutionize the regenerative medicine, offering unparalleled potential for tissue repair and replacement. Many studies indicated the contribution of iMSCs in the development of organoids, which are 3D structures and can mimic the organs structurally and functionally [20]. For instance, iMSCs generated with highly osteogenic, pro-angiogenic, and neurogenic capabilities were combined with a 3D bioprinting system to create shape-matched and functionally bioactive bone implants. These implants offer a promising strategy for addressing bone defects [21]. A 3D-printed hydrogel, loaded with human mesenchymal stem cells, played a crucial role in repairing a damaged uterine endometrium. This innovative approach not only restored the histological structures of the endometrium but also revived its ability to support embryo implantation and growth [22]. The efficacy of iMSCs was compared with primary MSCs derived from adipose tissue. The results indicated that iMSCs were comparable to adipose-derived MSCs in terms of ameliorating intestinal inflammation, increasing the intestinal Lgr5+ stem cells and promoting intestinal vascularization [23]. Additionally, many preclinical studies demonstrated that exosomes secreted by iMSCs have superior biological functions compared to MSCs, such as controlling the microenvironment and the secretion of bioactive paracrine factors [24]. The unique features of iMSCs such as unlimited availability, stable quality, high homogeneity, and relatively controllable and scalable production provide great advantages in regenerative medicine over the MSCs [25]. These properties of iMSCs not only increase the therapeutic applications of iMSCs for the treatment of conditions such as osteoarthritis, muscular dystrophy, skin diseases and cardiovascular disease, but also open a new horizon and an innovative tool within regenerative medicine.

## 6. Methods to Derive iMSCs from iPSCs

**Figure 2:**
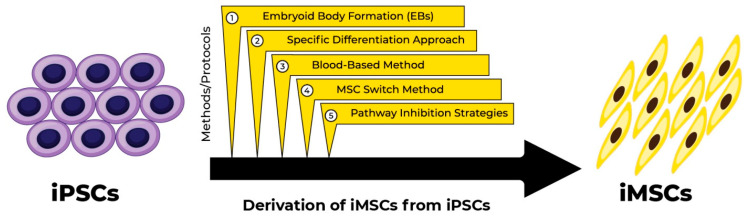
Methods of derivation of iMSCs from iPSCs. Induced mesenchymal stem cells can be derived from induced pluripotent stem cells with various methods including embryoid body formation, specific differentiation protocol, blood-based methods, MSC switch method, and pathway inhibitor strategies.

Considering the importance of iMSCs, many techniques have been employed to generate iMSCs from iPSCs (Figure 2). The common methods to produce iMSCs from iPSCs can be classified into five major categories: embryoid body formation (EBs), specific differentiation approach, blood-based method, MSC switch method, and pathway inhibition strategies. The comparison of these methods has been given in Table 1.

### 6.1. Embryoid Body Formation

Embryoid bodies (EBs) are three-dimensional (3D) clusters of cells. Usually these aggregates are obtained when pluripotent stem cells such as ESCs or iPSCs are cultured in vitro in suspension cultures [26]. This is one of the common methods that is employed to obtain iMSCs from iPSCs. First, iPSCs are enzymatically dissociated from their culture dishes, washed, and then maintained in a suspension culture under non-adherent conditions. Ultra-low attachment culture dishes are used to prevent cell adherence to the surface. Under these culture conditions, pluripotent cells aggregate to form embryoid bodies. The hanging drop method may also be used to produce embryoid bodies of controlled size. This method involves placing a drop of culture medium containing cells on a coverslip. The coverslip containing the cell suspension is then flipped over a specialized slide that has a depression in it. The drop hangs from the coverslip, sealed by the petroleum jelly to prevent evaporation of culture medium. This facilitates quick and homogeneous EB production by aggregating pluripotent stem cells through gravity. By carefully controlling the number of cells in each droplet, uniform EB size can be achieved [27].

iMSCs are derived from embryoid bodies by a series of processes, starting with the culturing of iPSCs in specific culture media. The medium consists of EB formation medium (1 mM L-glutamine), 80% knockout Dulbecco’s modified Eagle’s medium, 14 μM β-mercaptoethanol, 1% non-essential amino acids (NEAA), and 20% fetal bovine serum (FBS). Following the culturing of iPSCs, embryoid bodies are formed and culture medium is subsequently changed to generate iMSCs. Embryoid bodies undergo differentiation on the fifth day following their harvest. The EBs are cultured in suspension for eight days, before being transferred to the plates coated with gelatin for the growth of differentiated cells. The residual EB clumps are removed using a 40-μm cell strainer while the outgrown cells are transferred to the new flasks coated with gelatin. During this maintenance phase, cells are supplied with medium containing 10% FBS, KO-DMEM, 1% NEAA, and 1 mM L-glutamine. Flow cytometer analysis can be employed to identify the cell population based on the defined minimum criteria of iMSCs [2,28].

iMSCs derived from iPSC EBs hold considerable potential for regenerative medicine and disease modeling, offering a relatively simple and cost-effective approach. iMSCs obtained by embryoid body formation method have been found to promote new bone formation by increasing bone volume density [29]. However, there are a few challenges in applying this method, i.e., heterogeneity in EB size and shape, the difficulty in scaling up EB production, and lack of precise control over the microenvironment of embryoid bodies.

### 6.2. Specific Differentiation Approach

The specific differentiation method utilizes particular progenitor cells that are derived prior to culturing MSCs from iPSCs. This involves pre-differentiation of iPSCs into specific lineages, such as cardiac and neural progenitors. The rationale behind this method is based on the recognition that these progenitor cells share greater developmental and phenotypic similarities with MSCs than with iPSCs. Progenitor cells are intermediate cells, which provide a more efficient pathway for differentiation into MSCs in the respective medium. The MSC-specific differentiation media contain DMEM, 10–20% FBS, L-glutamine, essential growth factors, and various additives, such as antibiotics (penicillin-streptomycin) and ascorbic acid to enhance differential potential. This process allows the differentiation of iPSC-derived progenitor cells into mesenchymal cells [30]. For instance, the differentiation of iPSCs into cardiac or neural cells before further directing their differentiation into iMSCs offers a promising approach. This process can be improved by optimizing the method for iMSC production [31]. The differentiation of iPSCs generates into iMSCs through this method, which may have many advantages, such as the generation of a large number of cells and enhanced potential for proliferation, making them suitable for cell therapies. More specifically, the first step in synthesizing iMSCs by a specific differentiation method involves replacing the Geltrex coat with a gelatin-coated plate for iPSC culturing. This removal induces natural differentiation of iPSCs into mesenchymal progenitor-like cells in 5–7 days. Following the coating removal, the differentiating cells are trypsinized as single cells. These cells are then cultured in the media containing platelet-derived growth factor (PDGF), basic fibroblast growth factor (bFGF), and epidermal growth factor (EGF) for differentiation into MSCs, i.e., iMSCs. It takes about 10–20 days to convert the progenitors into iMSCs. The last step involves purifying the highly concentrated MSC-like cells and culturing them in a 96-well culture plate designed for single-cell growth. The single cell colonies consisting of MSC-like cells are identified. These colonies of iMSCs are further cultured in MSC media to obtain pure cultures of iMSCs. iMSCs produced with specific differentiation protocol show enhanced regenerative potential and thus are suitable for cell therapy [30]. However, a specific differentiation method is laborious, costly, and time-consuming. Additionally, this method involves a complex, multi-step process and requires the use of specific progenitor cells [29].

### 6.3. Blood-Based Methods

Blood-based method uses culture medium containing blood-based supplements to generate iMSCs. Blood based synthesis of iMSCs most frequently uses human platelet lysate (HPL). In this method, platelets are lysed to release growth factors and other proteins in solution. Human platelet lysate is prepared from buffy coat-derived platelet concentrates. Platelets are placed at −30 °C for 24 h and thawed at 37 °C to persuade lysis. Following the second freeze–thaw cycle, the platelet lysate is centrifuged to reduce the platelet fragments. Finally, the pooled units are thawed, aliquoted, and centrifuged at 3000× *g* for 30 min. The supernatant is kept at −80 °C until use. When required, aliquots are thawed at 37 °C in a water bath, and 2 IU/mL heparin is added to the thawed platelet lysate to prevent platelet aggregation during handling. The MSC culture medium is prepared by adding the appropriate concentration of platelet lysate to the complete culture medium. The culture medium is then supplemented with standard MSC growth factors (e.g., bFGF, IGF, or EGF). The platelet lysate method is a more useful method for generating iMSCs because more growth factors are released during the platelet’s lysis. Many important growth factors, such as insulin-like growth factor (IGF), transforming growth factor *beta* (*TGF-β*), and fibroblast growth factor (FGF), are vital in stem cell production. Moreover, this is an easy and low-cost method. This method helps in the large-scale production of iMSCs needed for clinical purposes according to good manufacturing practice. It also provides a xeno-free, human-based culture system for iMSC production, ensuring high proliferation with a low risk of heterogenic immune reactions. In addition to the above advantage, the platelet lysate method also faces certain challenges. This method is time-consuming and complex as it is a multistep process and strict storage requirements are needed. Despite centrifugation, some residual proteins or platelet fragments may remain in the lysate. These components could potentially trigger immune responses when used in clinical application.

### 6.4. MSC Switch Method

The MSC switch method involves changing or “switching” the iPSC culture medium with MSC growth media (i.e., DMEM/MEM/IMDM supplemented with FBS, bFGF, TGF- β1, Insulin, Transferrin, penicillin, phenol red, and serum) to cause spontaneous differentiation of iPSCs into MSCs. It is the least complex method of generating iMSCs [30]. In this method, iPSCs are obtained from different sources, and the iPSC culture media (KnockOut DMEM, KnockOut Serum Replacement (KSR), Activin A, LIF, TGF-β, Insulin, Vitamin C and Y-27632, B27 penicillin, and Phenol red) is replaced with the above mentioned MSC culture media. For example, adult peripheral blood mononuclear cells (PBMC) are used as an origin of cells for this method. These cells are cultured in the presence of five different factors (BCL-XL, OCT4, SOX9, MYC and KLF4) to reprogram the cells. This method effectively reprograms adult peripheral blood mononuclear cells into integration-free iMSCs, which have the potential to differentiate into three main cell types (trilineage differentiation) [32]. All the transcription factors that are added in the culture media have a specific role in reprogramming the iPSCs to iMSCs. As OCT4 and MYC play an important role in stimulating the early stage of reprogramming and maintaining pluripotency. SOX2 encourages mesodermal lineage commitment specifically towards MSC phenotype. KLF4 plays a role in maintaining stem cell state and supporting mesodermal differentiation while BCL-XL is known for preventing apoptosis and ensuring the survival of cells during the reprogramming process. Integration-free iMSCs are mesenchymal stem cells produced directly by reprogramming of somatic cells without the use of integrating vectors. Switch method is a simple and easy way of generating iMSCs [30]. FACS sorting helps in selecting a particular subpopulation, which results in more consistent results.

### 6.5. Pathway Inhibitor Method

The pathway inhibitor technique involves adding chemical inhibitors of certain pathways into the culture conditions to facilitate the differentiation of iPSCs into MSCs. Examples of these supplements are SB-431542(a TGF-β inhibitor), SB-203580 (a p38 MAPK inhibitor), and CHIR (a GSK-3 inhibitor). SB431542 inhibits the TGF-β signaling pathway, resulting in the downregulation of pluripotency genes and an increased differentiation into MSCs. SB-203580 inhibits p38 MAPK signaling pathway and enhances the stability of differentiating cells by mitigating cellular stress. CHIR (a GSK-3 inhibitor) combines with other inhibitors and provides an optimal environment for differentiation of MSCs. Pathway inhibitor protocols might become helpful in combination with the other described methods, thus enabling the development of rapid and more robust differentiation of iPSCs into iMSCs [30]. In this method, the iPSCs are cultured initially on Matrigel-coated plates using iPSC medium (basal medium). The medium is then substituted with MSC medium (culture medium) supplemented with the TGF-β pathway inhibitors for 14 days, allowing the conversion of iPSCs from epithelial cells to mesenchymal cells. After several passages, the cells are reseeded on uncoated dishes. On uncoated plates, cells adhere directly to the surface without the support of extracellular matrix proteins, thus identified as MSC [29]. Pathway inhibitor methods have an advantage of targeting specific signaling pathways that reduce heterogeneity. However, disadvantages of the pathway inhibitor method to produce iMSCs include labor-intensive, complex differentiation, and limited scalability.

## 7. Characterization of iMSCs

Minimum criteria for the characterization of primary MSCs was proposed by the International Society for Cellular Therapy (ISCT) in 2006. According to this criteria, primary MSCs have been characterized based on the following: (1) plastic-adherent growth, (2) positive expression of CD90, CD105, CD73, and negative expression of CD45, CD34, CD14 or CD11b, CD79α or CD19, and HLA-DR surface markers, and (3) differentiation into osteoblasts, adipocytes, and chondroblasts [33]. However, iMSCs derived from human iPSCs may require more rigorous characterization before their clinical applications. Choudhery et al. proposed the characterization of iMSC with additional parameters. This proposed minimum criteria for the characterization of iMSCs includes morphology, growth characteristics, surface marker expression, differentiation potential, and safety profile [2]. The above-mentioned criteria are a comprehensive set of parameters for defining iMSCs. Table 2 shows various parameters for characterization of induced mesenchymal stem cells.

### 7.1. Morphology

iPSC-derived mesenchymal stem cells, i.e., iMSCs, must have fibroblast-like, spindle-shaped morphology, which can be assessed by observing the cells under a microscope. iPSCs, which are round-shaped with prominent nucleoli, turn into spindle-shaped morphology during iMSC generation [2]. Therefore, the alteration of rounded morphology to spindle-shaped morphology exhibits the basis for apparent identification of iMSCs.

### 7.2. Plastic Adherence Growth

Similar to primary MSCs, iMSCs must exhibit plastic adherent growth under specific in vitro culture conditions. Plastic-adherent property is useful for isolating and purifying iMSCs by excluding the requirement of a feeder layer from the culture medium during iMSC generation [2].

### 7.3. Cell Surface Marker Expression

iMSCs must express CD29, CD44, CD73, CD90, and CD105. Additionally, iMSCs should exhibit negative expression of CD45, CD34, CD14 or CD11b, CD79a or CD19, and HLA class II (≤2%positive for iMSCs) cell surface markers. The percentage expression of the markers is determined by flow cytometry.

#### 7.3.1. Lack Expression of Pluripotent Induction Factors

iPSCs are generated by introducing the transcription factors such as Oct3/4 (Octamer-binding transcription factor 3/4), Sox2 (SRY-box transcription factor 2), c-Myc (Myelocytomatosis oncogene), Klf4 (Kruppel-like factor 4) in somatic cells [34]. These transcription factors enhance the pluripotency of these cells. In differentiating iPSCs, the expression of these pluripotent markers starts to decline. When fully differentiated, iMSCs must lack the expression of these pluripotency markers, showing reduced pluripotency of iPSCs and their enhanced differentiation into iMSCs [2]. Quantitative PCR is used to quantify the expression of pluripotent genes. Firstly, RNA is extracted from iMSCs, and cDNA is made by reverse transcription. cDNA is then amplified, and the expression profile of the above-mentioned genes is analyzed.

#### 7.3.2. Teratoma Formation Assay

Teratoma is a benign tumor derivative of three germ layers (mesoderm, ectoderm, and endoderm). Teratoma formation assay is a standard method to identify the pluripotency of cells. iPSCs have pluripotency potential and thus can form cells derived from three germ layers. iPSCs thus can form teratomas when injected in immunocompromised mice. The assay involves the transplantation of cells in immunocompromised mice to analyze their pluripotency status. Unlike iPSCs, MSCs are multipotent and can only differentiate into a limited number of cell types. Thus, iPSC-derived iMSCs must lose the ability for teratoma formation [2]. The ability to lack teratoma formation can be confirmed by transplanting iMSCs in solid organs such as testis, muscles, or in subcutaneous injections into immune-deficient mice. The absence of teratoma formation confirms the differentiation of iPSCs into iMSCs [2].

#### 7.3.3. Secretome Analysis

Similar to MSCs, iMSCs should also release paracrine factors such as VEGF, IGF, SDF-1, PDGF, and FGF. The release of these factors is important, as MSCs exert their therapeutic effects not only by trans-differentiation into respective cell types but also by secreting a number of growth factors, cytokines, and interleukins. The confirmation of the paracrine status of MSCs obtained from iPSCs is important in that it ensures the therapeutic potential of iMSCs. For this purpose, a combination of different growth factors, cytokines and interleukins can be adopted [35].

#### 7.3.4. Differentiation Assays

When cultured under appropriate conditions, iMSCs must demonstrate the ability to differentiate into osteoblasts (bone cells), adipocytes (fat cells), and chondroblasts (cartilage cells). This is also one of the criteria for identification of primary MSCs. Differentiation assays are used to confirm the multipotency of iMSCs by inducing differentiation into adipogenic, osteogenic, and chondrogenic lineages. MSCs can be induced to differentiate into osteogenic lineage by culturing them in osteogenic induction medium (α-MEM supplemented with FBS (10%), L-ascorbic acid 2-phosphate (100 μM), L-glutamine (2 mM), dexamethasone (10 nM), β-glycerophosphate (2 mM), penicillin (100 U/mL), and streptomycin (100 μg/mL) [36]. Osteogenic differentiation is assessed by Alizarin Red S staining which detects calcium deposits in differentiating cells and tissues. Von Kossa staining is also used to confirm the osteogenic differentiation with silver nitrate solution. This is an indirect assay that replaces the calcium ions with silver ions. When silver nitrate is exposed to light, it turns to black metallic silver. MSC-derived osteogenic cells show positive reaction with Von Kossa staining. MSCs are induced to form chondrocytes by culturing them in chondrogenic medium, consisting of Dulbecco’s Modified Eagle’s Medium (DMEM), supplemented with 10% ITS+ premix tissue culture supplement, 1% sodium pyruvate, 10−7 M dexamethasone, 1 μM ascorbate-2-phosphate, and 10 ng/mL human transforming growth factor-beta 1 (TGF-β1) [37]. The chondrocyte differentiation potential of iMSCs is determined by Alcian blue staining, which identifies proteoglycans in the extracellular matrix. The differentiation of iMSCs into adipocytes follows the culturing of these cells in adipogenesis media containing glucose (4.5 g/L), 3-isobutyl-1-methylxanthine (0.5 mM), dexamethasone (1 μM), indomethacin (0.5 mM), and insulin (10 μg/mL) [36]. Adipogenic potential of iMSCs is determined by Oil Red O staining, which identifies lipid droplets.

## 8. Applications of iMSCs in Regenerative Medicine

**Figure 3:**
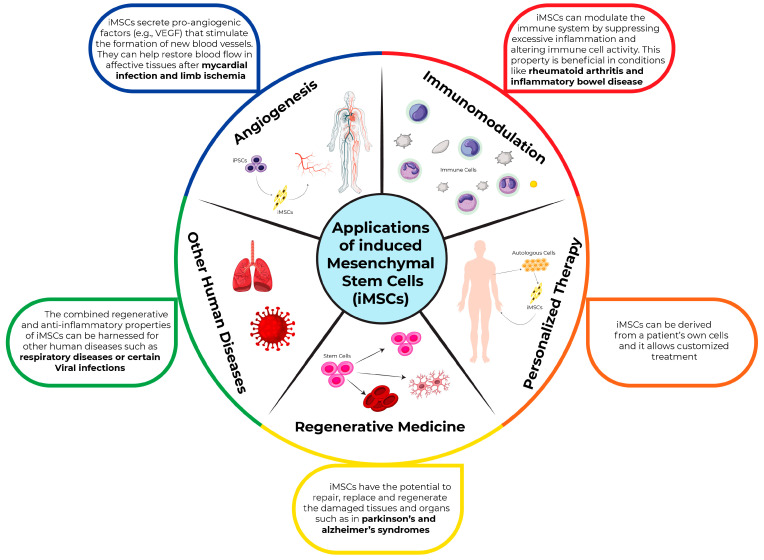
Application of induced mesenchymal stem cells. iMSCs have many applications in regenerative medicine, personalized therapy, immunomodulation, angiogenesis, and to treat other human diseases.

iMSCs have a wide range of therapeutic applications. iMSCs represent a non-exhausted, readily available, autologous source of stem cells with equivalent or superior properties to those of the primary MSCs. These characteristics make iMSCs an ideal type of cell for personalized medicine. Similar to the primary MSCs, iMSCs can be employed to cure various types of diseases (Figure 3). iMSCs may offer superior regeneration compared to conventional methods, including the utilization of adult stem cells. iMSCs are potentially a reliable source of cells in regenerative medicine due to their extensive proliferative abilities, multipotency, and reduced ethical concerns compared to ESCs and even adult stem cells isolated from primary tissues. iMSCs have potential to treat various diseases including degenerative diseases, Osteoarthritis, Macular Degeneration, Amyotrophic Lateral Sclerosis (ALS), Chronic Obstructive Pulmonary Disease (COPD), Muscular Dystrophy, skin diseases, cancer, and Spinal Muscular Atrophy. iMSCs have shown promising results in animal models of osteoarthritis, where they have contributed to the repair of cartilage and reduction in inflammatory markers [38]. Additionally, the immunomodulatory properties of iMSCs make them an attractive candidate for treating autoimmune disorders, as they can modulate the immune response and reduce inflammation [39].

Interestingly, iMSCs can also be used as acellular therapy as they are a potential source of exosomes. Exosomes can be derived from iMSCs through a systematic process. At first, iMSCs are cultured under specific conditions that promote their growth and exosome secretion. After a predetermined incubation period, the culture medium containing the exosomes is collected. The resulting exosomes are then isolated by using methods such as ultracentrifugation or size-exclusion chromatography. Like other sources of exosomes, iMSC-derived exosomes are less likely to provoke an immune response because they lack the complete cellular structure that could be identified as foreign. Moreover, exosomes retain many of the beneficial paracrine signaling properties of their parent cells, providing therapeutic effects such as anti-inflammatory responses and tissue regeneration without the complications linked to cell therapies. Their small size allows better tissue penetration, which enhances their therapeutic delivery. Exosomes are also more stable and easier to store, as they can be cryopreserved and reconstituted without losing functionality. Exosomes reduce the risk of vascular blockage, making them a safer option for systemic administration. A study by Kim et al. indicated that iMSC-derived exosomes promote the growth of skin cells. These exosomes stimulate the phosphorylation of extracellular signal-regulated kinase (ERK1/2), which significantly enhances the proliferation of human skin cells and dermal fibroblasts. This increased activity is associated with higher levels of collagen and fibronectin secretion, which are essential for skin regeneration. A recent review discussed the role of iMSC-derived exosomes in tissue regeneration, connective tissue repair, regeneration of bones, cartilage, and skin. Exosomes improved wound healing, stimulated bone regeneration, and enabled cartilage repair by transferring proteins, mRNAs, and microRNAs [40]. iMSC exosomes can also stimulate epithelial cell proliferation and migration, which is called re-epithelialization. This is a key healing process in chronic wounds. Although, it is important to note that pre-clinical studies show promising potential outcomes in the use of iMSC-derived exosomes for regenerative purposes. However, clinical use of iMSC-derived exosomes is not an active practice at this moment because of a few scalability issues with exosomes. These include limitations in purity, integrity and yields of exosomes [40].

### 8.1. Bone Regeneration

iMSCs have been reported to possess osteogenic potential and, therefore, could form bony structures in scaffolds [41]. Transplantation of iMSCs was shown to effectively promote bone repair and angiogenesis in the necrotic region of the femur [42]. iMSC-derived exosomes secrete cytokines, growth factors, mRNAs, and miRNAs, which increases the proliferation and migration of bone marrow-derived MSCs, hence significantly preventing bone loss [43,44]. iMSCs are also potentially capable of periodontal regeneration. Upon transplantation into periodontal tissues, human-induced mesenchymal stem cells facilitate the formation of new mineralized tissues. This leads to significant improvements in periodontal regeneration, promoting healthy gum growth and repair [45].

### 8.2. Cartilage Regeneration

Cartilage is crucial for the strength and normal movement of joints. It is damaged with age and other factors such as injuries, inflammation, etc. To repair cartilage damage, iMSCs have been shown to be effective. One of the pioneering studies in this context showed that BMP2 treatment of iMSCs enhanced the production of cartilage-specific type II collagen. This collagen is part of the articular cartilage, which causes osteoarthritis upon degradation. Osteoarthritis is a degenerative joint disease where cartilage breaks down, causing pain and stiffness in the joints, which results in movement problems. It is the most common arthritis condition, which occurs mainly in elder people. iMSCs possess significant chondrogenic differentiation potential, making them capable of cartilage regeneration [46]. iMSC-derived exosomes can increase the collagen II production, decrease proteoglycans production in extracellular cartilage matrix (ECM), and increase the migration of chondrocytes, thereby increasing the therapeutic efficacy in osteoarthritis [24,38].

### 8.3. Kidney Diseases

Chronic kidney disease (CKD) is the gradual loss of kidney function over time due to renal fibrosis. iMSCs are reported to prevent kidney fibrosis by SIRT6/β-Catenin Signaling Pathway [47]. Intravenously administrated iMSCs protect the kidney against CKD injury in CKD parenchyma [48]. iMSCs are also able to protect kidneys from ischemia reperfusion [49]. Additionally, iMSC-derived exosomes decrease inflammatory reactions and improve renal function by increasing SIRT6 levels and decreasing the levels of β-catenin [47].

### 8.4. Cardiovascular Disorders

Cardiovascular disorders such as ischemic heart disease, myocardial infarction, and heart failure may benefit from iMSC-derived exosomes, as they promote angiogenesis, reduce cardiomyocyte apoptosis, and enhance myocardial repair [50]. Additionally, conditions such as dilated and hypertrophic cardiomyopathy, cardiac fibrosis, and diabetic cardiomyopathy could be addressed through their anti-fibrotic effects and oxidative stress reduction [51]. iMSC-derived exosomes hold significant potential for treating cardiac disorders due to their regenerative and anti-inflammatory properties. Their mechanism involves the delivery of bioactive molecules, including microRNAs (e.g., miR-21, miR-22), which regulate inflammation, apoptosis, and tissue remodeling, along with their ability to modulate immune responses and enhance endogenous repair pathways [52]. iMSC-derived extracellular vesicles reduce age-associated vascular endothelial dysfunction and improve arterial aging. Additionally, these extracellular vesicles can also reduce arterial tension and the symptoms of hypertension [53]. While these applications are promising, they are under active investigation, with ongoing studies to support the idea of their clinical efficacy and safety.

### 8.5. Neurological Disorders

Neurodegenerative disorders are chronic conditions that progressively weaken and destroy components of the nervous system, particularly the brain cells. These disorders originate from the continuous deterioration of cells and neural connections vital for coordination, mobility, strength, cognition, and sensation. Alzheimer’s is a progressive neurodegenerative disorder, which primarily affects old adults, leading to memory loss (dementia), and other behavioral changes. It is characterized by the accumulation of amyloid-beta plaques and tau tangles in the brain. It results in neuronal damage and impaired synaptic connections. Symptoms typically start with mild memory issues and escalate to severe cognitive impairment. While there is no cure to this disease, treatments focus on managing symptoms, and ongoing research is aimed at understanding this disease better and to develop effective therapies. iMSC-derived exosomes reduce amyloid-beta production. iMSC-exosomes release miR-146a that acts on astrocytes to reduce NF-Kb signaling and reduce neuronal impairment. miR-146a also acts on hippocampal neurons to increase synaptic transmission. These effects collectively contribute to decreasing the symptoms of Alzheimer’s disease [24].

Parkinson’s disease, another neurodegenerative disorder, involves the degeneration of dopamine-producing neurons in the brain. This is particularly in the substantia nigra region of the brain. The loss of dopamine leads to tremors, rigidity, slow movement, and unstable posture. iMSC-derived exosomes are reported to increase dopamine production by delivering neurotropic factors such as BDNF and GDNF, promoting the survival and differentiation of dopaminergic neurons. In addition, these exosomes also modulate neural inflammation and transfer functional RNAs that enhance dopamine synthesis. Additionally, they protect mitochondrial function in neurons, supporting dopamine production. Hence, dopaminergic neurons are prevented from being lost and their apoptosis is also hindered, resulting in reduced symptoms of Parkinson’s disease [24].

### 8.6. Personalized Therapy

Personalized therapy, often referred to as personalized medicine or precision medicine, is a healthcare approach that tailors the medical treatment to the specific individual characteristics and needs of each patient. A patient’s genetic profile mainly guides decisions regarding prevention, diagnosis, and treatment of diseases. That is quite achievable by using iMSCs because iMSCs are obtained from the patient’s own iPSCs, which can minimize the risk of therapeutic rejection. A recent study has demonstrated that urine-derived iPSCs from brain tumor patients can differentiate into iMSCs for use in the same patient [54]. iMSCs are significantly more advantageous than MSCs derived from organs or tissues because iMSCs originate from a cell source that has a tremendous proliferative potential (i.e., iPSCs). This eliminates the source and availability limitations. Furthermore, tissue-derived primary MSCs exhibit significant heterogeneity, which can affect their therapeutic results. Conversely, iMSCs are potentially more uniform as they can be generated from a single clone of iPSCs. Therefore, it is anticipated that iMSCs will produce more reliable and consistent outcomes, even when different batches of iMSCs are compared. Similarly, the administration of iMSCs directly into the brain showed more powerful protective effects on the nervous system compared to MSCs produced from the umbilical cord in a rat model with reduced oxygen and blood supply to the brain [55]. Several studies have reported that iMSCs, including those derived from urine, exhibit enhanced therapeutic efficacy in experimental disease models. For example, intra-myocardial administration of iMSCs resulted in better regeneration compared to BM-MSCs in the animal model of heart failure [56].

### 8.7. Immunomodulation

Immunomodulation is the process of changing or controlling the immune system’s reaction. Depending on the therapeutic objective, it requires modifying the immune system’s function to either increase or decrease its activity. When it comes to cell treatment, the immune system always acts as a barrier, because it elicits an immune response against the alloantigens present on the graft. iMSCs downregulate the expression of pluripotency genes and suppress T cell proliferation and dendritic cell activation [57]. By impairing functions of dendritic cells, iMSCs reduce their ability to present antigens and activate T cells. This suppression can be beneficial in preventing graft rejection, autoimmune diseases, or excessive inflammation, but it may also weaken the immune system’s capability of combating infections or tumors. Hence, a diminished immune response and a more tolerant immune environment is maintained [58]. iMSCs are ideal for cell-based immunotherapy because they can be made patient specific and hence escape immune rejection. Researchers found that iMSCs could inhibit NK cells proliferation and disrupt their cytolytic machinery, thereby preventing tissue rejection in the recipient. As a result of their immunomodulatory properties, iMSCs are potentially good candidates for prophylactic treatment in graft versus host diseases (GVHD) [59]. Usually, during transplantation, circulating natural killer cells target and destroy the graft [60]. The research conducted by Giuliani et al. has demonstrated that when human iMSCs are cultured in a controlled environment, they significantly reduce the cytolytic capabilities of NK cells. Therefore, iMSCs can be considered a valuable therapeutic choice for the prevention of allograft rejection [59].

### 8.8. Angiogenic Potential

The angiogenic potential of iMSCs refers to their ability to stimulate the formation of new blood vessels, which is crucial for tissue repair and regeneration. iMSCs have a strong capacity to promote angiogenesis by releasing growth factors that encourage endothelial cell proliferation and organization into vascular structures. This potential makes iMSCs particularly promising in therapeutic contexts, such as enhancing blood flow in ischemic tissues and improving wound healing processes. For example, ischemic diseases, which are characterized by inadequate blood or oxygen supply to a specific organ, i.e., the heart, are treatable by using iMSCs. iMSCs are emerging as a promising therapeutic approach for treating myocardial and limb ischemia [61]. A recent investigation by Arakawa et al. has shown that iPSC-derived iMSCs attenuate cerebral ischemia-reperfusion injury. The achievement was made possible due to the ability of iMSCs to suppress inflammatory signals and oxidative stress in a rat model of transient middle cerebral artery blockage [62]. Another study revealed that intramuscular injection of iMSC-derived exosomes significantly increased arterioles, venules, capillary density (Micro vessel density), and blood perfusion in mice ischemic limbs, indicating a reduction in ischemia injury [63].

Furthermore, iMSC-derived exosomes potentially contribute significantly to angiogenesis by increasing the levels of molecules associated with new blood vessel formation, such as vascular endothelial growth factor (VEGF) [64]. These may elevate the expression of angiogenesis-related molecules and facilitate the migration, proliferation, and tube formation of human umbilical vein endothelial cells (HUVECs) [63]. This supports the idea of their potential application in therapies aimed at tissue regeneration and vascular repair. A comprehensive understanding of the pathological mechanism is required for the advancement of therapeutic drugs to treat a wide range of diseases. In vitro models that represent in vivo development are highly valuable for the formulation of these drugs. Nevertheless, the limited availability of human tissues and the absence of animal models have hampered the research on human diseases and drug screening.

## 9. Disease Modeling and Drug Discovery

A complete understanding of the biological mechanisms is necessary for the development of treatments to cure a variety of disorders. In vitro models that mimic in vivo development are useful for the discovery of new curative drugs. However, the limited availability of human tissues and the lack of animal models have made it difficult to conduct pharmacological screening and study human diseases. iMSC technology offers new opportunities for modeling human diseases and manufacturing drugs for disease treatment. iMSCs are ideal for disease modeling and drug discovery because they closely resemble human tissues, are easily accessible, and can be differentiated into various cell types. This makes them a powerful tool for studying disease mechanisms, screening potential drug candidates, and developing personalized treatments. However, scientific research on human genetic diseases and drug screening is limited due to the inaccessibility of tissues and the absence of appropriate animal disease models [65].

The primary function of iMSCs in disease model studies is to promote tissue repair and modulate immune responses, which is analogous to the function of tissue-derived MSCs. However, the exact roles of iMSCs may differ in different disease conditions. iMSCs promoted periodontal regeneration in a rat model of periodontitis [45]. It was discovered that iMSCs enhanced mucosal healing mechanisms in a mouse model of inflammatory bowel disease (IBD) via producing TSG-6. Mice showed improved colonic mucosa repair after receiving administered iMSCs. Enhanced numbers of CD44+ Lgr5+ cells and higher proliferation of epithelial cells were the hallmarks of this improvement [66]. Another study on a mouse model showed that iMSCs primarily work by suppressing the immune system. Plaque size was significantly reduced after 12 weeks of intravenous iMSC administration in ApoE mutant mice fed a high-fat diet. The inflammation of arterial sclerosis (AS) was reduced after the infusion of iMSCs because serum inflammatory cytokines, especially TNFα and IL6, were reduced [67]. Th17 and Treg were upregulated, cytokines for Th1 and Th2 were downregulated, and T cell proliferation was suppressed after iMSC implantation [68]. In addition, research has demonstrated that iMSCs can substantially reduce the growth, activation, and differentiation of cytotoxic CD8 T cells into Tc1 cells and CD8 T cells producing IL17 [69]. Whether mitochondrial transfer from iMSCs can maintain the survival of retinal ganglion cells (RGCs) and restore retinal function is currently being investigated. Injecting iMSCs into the retina of Ndufs4 mutant mice significantly increases the survival rates of RGCs, which is correlated with the observed improvements in retinal function. The loss of RGCs caused by mitochondrial damage can be prevented by transferring functional mitochondria to RGCs through the vitreous after iMSC transplantation [70]. Mice with asthmatic inflammation showed a significant decrease in T helper 2 cytokines and an improvement in epithelial cell mitochondrial dysfunction after receiving iMSC transplantation. Both in vitro and in vivo observations in mice showed that the iMSCs facilitated mitochondrial transfer by forming tunneling nanotubes (TNTs) with epithelial cells [71]. Restoring functional cells through paracrine signaling or mitochondrial transfer appears to be the primary mechanism by which iMSCs restore function in disease treatment.

The development of new treatments for hereditary disorders depends on our ability to better understand the pathogenic mechanisms that underlie these conditions. Research on these inheritable diseases is still problematic due to restrictions in patient tissue and an absence of suitable animal models. New insights into these diseases can be acquired through the use of cellular disease models that incorporate patient-specific iPSCs. The optimal instruments for pathologic research and platforms for drug screening and toxicity testing are iMSCs differentiated from patient iPSCs [65]. A rare hereditary disorder known as fibrodysplasia ossificans progressive (FOP) causes connective tissues to undergo progressive heterotopic ossification (HO). Mutations in the ACVR1 gene, which encodes Activin-A receptor type 1, are responsible for the development of this condition. This protein is integral to the bone morphogenetic protein (BMP) pathway. Recombinant induced pluripotent stem cells (iPSCs) originating from FOP patients and rescued iPSCs (resFOP-iPSC) were produced by our group [72]. iMSCs were derived from both iPSC clones via the neural crest cell lineage. FOP-iMSCs demonstrated increased chondrogenic capacity and increased activation of the SMAD1/5/8 pathway relative to resFOP-iMSCs, effectively replicating the disease phenotype. We discovered a unique FOP pathway by using these cells to evaluate TGFβ superfamily ligands that might specifically stimulate BMP signaling through FOP-ACVR1 using a luciferase reporter (BRE-Luc) assay. The activation of BMP signaling is induced by Activin-A, a TGFβ signal transducer, through FOP-ACVR1. The skeletal muscle of immunodeficient mice was implanted with FOP- and resFOP-iMSCs together with C3H10T1/2 cells that expressed Activin-A in order to create an in vivo evaluation system. The development of HO at the transplant site six weeks later suggests that Activin-A stimulates the creation of extra skeletal bone in FOP [73].

A high-throughput screening (HTS) method was set up utilizing FOP-iMSCs to uncover novel therapeutic targets and to reveal the molecular processes of the increased chondrogenesis induced by Activin-A. The mTOR signalling pathway was found to have a significant role in the excessive chondrogenesis observed in FOP-iMSCs, according to the screening of around seven thousand small-molecule compounds. The HO gene was significantly downregulated in FOP-iMSC transplanted mice when the widely available mTOR inhibitor, rapamycin, is administered. Activin-A upregulates the chondrogenesis activity of FOP-iMSCs, and a DNA microarray experiment suggests that ENPP2 (autotaxin) acts upstream of mTOR signalling. The FOP study demonstrates that drug discovery platforms and hereditary disease models based on iMSCs can accurately mimic disease phenotypes and provide a significant advantage in studying potential drugs and important disease pathways, which can speed up the process of developing new treatments [74].

The pathophysiology was studied by Zhang et al. using iMSCs produced from individuals with Hutchinson–Gilford progeria syndrome (HGPS). The segmental premature ageing illness known as HGPS is caused by progerin, a variant of Lamin A that is shortened and farnesylated. It damages mesenchymal lineages. DNA damage, anomalies in nuclear shape, and the presence of calponin-staining inclusion bodies are all characteristics shared by fibroblasts obtained from HGPS patients, and these are also observed in HGFP-iMSCs. Hypoxia in particular was found to impair the viability of HGFP-iMSCs in both laboratory and animal studies. The ability of HGPS-iMSCs to withstand hypoxia was restored when shRNA reduced progerin levels. Scientists have postulated that the MSC pool is depleted due to the replacement of lost mesenchymal tissue, as HGPS-iMSCs become excessively sensitive to their hypoxic microenvironment due to progerin toxicity [75]. Cicero et al. used alkaline phosphatase activity (ALP) monitoring to conduct a high-throughput screening of 2800 small compounds that might prevent HGPS-iMSCs from differentiating into osteogenic lineage. Progerin expression was substantially reduced by four of the seven compounds that were identified as significantly reducing premature osteogenic differentiation [76].

Several disease models have been used to compare iMSCs with MSCs produced from tissues. In a chronic pulmonary disease (COPD) animal model, iMSCs mitigated cigarette smoke-induced lung damage better than BM-MSCs. Lung tissues showed an imbalance between proliferation and apoptosis. iMSCs considerably reduced CS-induced COX2 and CINC1 levels, neutrophil, and macrophage infiltration [77]. Using an inflammatory bowel disease (IBD) model, the therapeutic effectiveness of BM-MSCs, UBB-MSCs, ESC-MSCs, and iMSCs was evaluated. UCB-MSCs and BM-MSCs both considerably reduced inflammation, although ESC-MSCs and iMSCs showed slightly lesser anti-inflammatory effects [78]. In addition, using a mouse model of IBD, another study compared the effectiveness of iMSCs and AD-MSCs. iMSCs and AD-MSCs both promoted intestinal vascularization, increased the number of intestinal Lgr5+ stem cells, and considerably reduced inflammation in the intestines [23]. Limited research has compared iMSCs to MSCs produced from tissues. However, both types of MSCs demonstrate comparable therapeutic efficacies. The secretome of iMSCs may have more therapeutic promise because of their low heterogeneity and ease of growth.

## 10. Limitations and Future Direction

iMSCs have the potential to overcome the limitations of primary MSCs. However, several factors, including differentiation capacity, epigenetic memory, cell origin and derivation methods, quality and efficiency, immature differentiation potential, lack of understanding of MSC stemness/potency, and challenges in generating clinical-grade iPSC-MSCs, must be considered before iMSCs can be utilized in clinical settings. One major concern is tumorigenicity, as iMSCs may develop into tumors after transplantation due to various factors, including donor age, host tissue, growth regulators, and mechanisms stimulating MSC activity. The differentiation complexity of iMSCs also presents challenges, as they require multiple differentiating steps, strict quality control, and molecular profiling to ensure retention of intended phenotypic, functional, and safety profiles. The differentiation capacity of iPSC-MSCs is a key characteristic for therapeutic use, but little is known about the distinctions between iPSC-MSCs from different sources. Despite expressing MSC surface antigens, iPSC-MSCs produced using diverse procedures are less able to differentiate than BM-MSCs, particularly in adipogenesis. Differentiation techniques affect hPSC-MSC quality and efficiency. Moreover, differentiated iPSC-MSCs have a potential to return to their parent cell type, suggesting that iPSCs could be affected by incomplete reprogramming or epigenetic memory [79].

The applications of iMSCs in regenerative and personalized medicine hold significant promise. However, their use also presents certain limitations, particularly when it comes to the timing of treatment and the choice between autologous and allogeneic cells. Autologous iMSCs derived from the patient’s somatic cells generate iPSCs but require considerable time for cell harvesting, reprogramming, and expansion, which makes them an impractical approach for use in acute conditions such as myocardial infarction. However, allogeneic iMSCs can be prepared in advance from other donor cells, for example, donor fibroblast or blood cells, and made readily available for immediate intervention. While allogeneic iMSCs offer the advantage of immediate availability, they may carry the risk of immune rejection. Therefore, the choice between autologous and allogeneic iMSCs should be carefully made, taking into consideration the particular clinical scenario, the urgency of treatment, and the potential benefits and risks associated with both approaches [80].

The immune system and inflammation can be modulated by iMSCs. Autologous iPSC-MSCs produced from patients are preferable to allogeneic iMSCs for regenerative medicine due to their reduced immune response [79]. In comparison to primary MSCs, iPSC-MSCs have a longer life span, faster proliferation, and more reliability and homogeneity in cell supply. However, they do not possess immunosuppressive properties and have an immature differentiation capacity. Further research is required to determine the internal and environmental characteristics that distinguish iPSC-MSCs from native MSCs [79]. Lack of knowledge of the mechanism behind MSC stemness or potency provides another significant barrier to the clinical application of iPSC-MSCs. Future research is necessary on biomarkers or regulators of iMSC stemness/potency employed in therapeutic settings [81].

Strict quality control, including molecular profiling, functional assays, and safety testing to avoid undesired differentiation or tumorigenic potential, is necessary to guarantee that iMSCs retain their intended phenotypic, function, and safety profile [16]. Future research directions for iMSCs should include preconditioning and modulation to enhance their survival, function, and therapeutic effects in patients. This can be achieved by pre-exposing cells to toxins, chemicals, and inflammation to focus on their immunomodulatory and regenerative capabilities [16]. Another promising area is CRISPR-Cas9-based manipulation of iMSCs. Advanced gene editing tools, such as CRISPR-Cas9, have shown effectiveness in modifying iPSC processes, such as correcting genetic defects or introducing specific genetic modifications. Mutations associated with specific diseases can be introduced into cells to investigate the efficacy of various drugs or disease models. It can be employed successfully for corrective purposes, such as genetic mutation repair or the deletion of undesirable genomic variations [82,83]. Editing the genome is a powerful strategy that can be used to improve the genetic stability of induced mesenchymal stem cells and make them appropriate for regenerative medicine [84].

## 11. Conclusions

The study and development of iMSCs mark a significant advancement in the field of regenerative medicine. iMSCs exhibit prominent advantages over MSCs, as they are derived from iPSCs confronting major challenges associated with MSCs. The limitations associated with MSCs are low number of MSCs in adult donor tissues, cell-source related variability, the negative impact of donor-age on their regenerative potential, limited growth potential, and early senescence in in vitro cultures. The potential to produce iMSCs through several methods and protocols, such as embryoid body formation, specific differentiation method, platelet lysate method, switch method, or pathway inhibitor method, has provided the foundation for the standardized and reproducible generation of iMSCs. The minimum criteria for defining iMSCs include a comprehensive list of parameters including morphology and growth characteristics, surface marker expression, differentiation potential, functional profile, and safety profile. These cells have remarkable regenerative, immunomodulatory, and angiogenic potential, which make them potent and effective therapeutic agents for the treatment of many diseases. iMSCs may mark a significant advancement in regenerative medicine, providing solutions to many of the limitations associated with MSCs. The versatility of iMSCs, combined with advancement in iPSC technology, establish a strong base for developing new therapies for a wide range of injuries and degenerative diseases. However, to fully utilize the therapeutic potential of iMSCs, further research is important to overcome the related challenges, including the establishment of methods for uniform production, extensive safety assessments, and the formation of regulatory frameworks to guide their clinical use. With continued advancement, iMSCs could play a crucial role in the future of regenerative medicine, offering hope for patients with previously untreatable conditions.

## Figures and Tables

**Table 1:** iMSC generation: Comparison of different methods.

Method Name	Yield	Time	Advantages	Disadvantages
**Embryoid body formation**	High	4–5 weeks	Simple, cost effective, potential for regenerative medicine and disease modeling	Lack of precise control over microenvironment, heterogeneity in EB size and shape
**Specific differentiation**	Moderate to high	3–4 weeks	Reduces heterogeneity, more regenerative potential	Complex, time consuming, expensive
**Blood based method**	High	3–4 weeks	large-scale production, reduces risk of heterogenic immune reactions	Commercial distribution of hPL (human platelet lysate) is rare, contamination risk the use of human-derived products raises ethical issues that must be reviewed
**MSC switch**	Relatively high	2–3 weeks	Generates MSCs in 2–3 weeks, MSC properties can be controlled by use of particular culture media	Requires expertise, May raise concern about safety and genetic stability in clinical setting
**Pathway inhibition**	Variable	2–3 weeks	Less differentiation time then traditional methods, reduces heterogeneity	Labor intensive, complex differentiation, limited scalability

**Table 2:** Characterization of induced mesenchymal stem cells.

Parameters	Characteristics
Morphology	Spindle-shaped fibroblast-like cells
Growth	Plastic adherence
Cell surface marker	Positive markers: CD29, CD44, CD73, CD90, and CD105 Negative markers: CD45, CD34, CD14 or CD11b, CD79a or CD19, and HLA class II.
Pluripotency markers	Lack expression of relevant pluripotency markers e.g., Oct3/4, Sox-2, and c-Myc, KLF4
Teratoma formation assay	Lack ability to form teratoma in immunocompromised mice
Secretome	Secretes MSC specific growth factors such as VEGF, IGF, SDF-1, PDGFs, FGF, etc.
Differentiation potential	Multilineage differentiation: adipogenic, osteogenic, and chondrogenic lineages

## Data Availability

Not applicable.

## Abbreviations

AMs: Alveolar macrophages; bFGF: Basic fibroblast growth factor; BCL-XL: B-cell lymphoma-extra-large; CDKN2C: Cyclin-dependent kinase inhibitor 2C; CHIR: CHIR99021; c-Myc: Myelocytomatosis oncogene; CRISPR-Cas9: Clustered regularly interspaced short palindromic repeats-associated protein 9; DM: Diabetes mellitus; DNP: Diabetic polyneuropathy; EBs: Embryoid bodies; EGF: Epidermal growth factor; Epha4: Eph receptor A4; ESCs: Embryonic stem cells; Exo: Exosomes; FACS: Fluorescence-activated cell sorting; FoxO1: Forkhead box O1; GSK-3: Glycogen synthase kinase-3; GVHD: Graft versus Host Diseases; H-NSCs: Hippocampal neural stem cells; HGPS: Hutchinson–Gilford progeria syndrome; HPL: Human platelet lysate; HSCs: Hematopoietic stem cells; HUVECs: Human umbilical vein endothelial cells; iMSC-sEV: Induced mesenchymal stem cell-derived extracellular vesicles; iMSCs: Induced mesenchymal stem cells; iPSCs: Induced pluripotent stem cells; ISCT: International Society for Cellular Therapy; KLF4: Krüppel-like factor 4; KO-DMEM: Knockout Dulbecco’s modified Eagle’s medium; MAPK: Mitogen-activated protein kinase; miR-21-5p: MicroRNA-21-5p; miR-486-5p: MicroRNA-486-5p; MYC: Myc proto-oncogene protein; NEAA: Non-essential amino acids; NK cells: Natural killer cells; Oct3/4: Octamer-binding transcription factor 3/4; OCT4: Octamer-binding transcription factor 4; OI: Osteogenesis imperfecta; PBMC: Peripheral blood mononuclear cells; p38 MAPK: p38 mitogen-activated protein kinase; POCD: Postoperative cognitive dysfunction; SB: Small molecule inhibitor (related to SB-431542, SB-203580); Sox2: SRY-box transcription factor 2; SOX9: SRY-box transcription factor 9; TGF-β: Transforming growth factor-beta; tGCI/R: Transient global cerebral ischemia/reperfusion; WJSC: Wharton’s jelly stem cells.
